# Conformational Entropy Contributions to the Glass Temperature of Blends of Miscible Polymers

**DOI:** 10.6028/jres.102.018

**Published:** 1997

**Authors:** Hans Adam Schneider

**Affiliations:** Freiburger Material-forschungszentrum, FMF, der Universität, Stefan-Mejer-Strasse 21, D-79104 Freiburg, Germany

**Keywords:** concentration power equation, conformational entropy, flexible bond, free volume, glass transition, interchain orientation, miscible polymer blends

## Abstract

Because of negligible contributions of combinatorial entropy, miscibility of polymers is attributed predominantly to favorable (exothermic) enthalpic effects of mixing, i.e., to strong interactions between the blend components, which have to overcome the cohesive forces acting within the components. Miscibility of amorphous polymers usually is associated with the presence of a single glass temperature of the blend. Although stronger hetero-contact interactions are thermodynamically required for polymer miscibility, the majority of miscible binary polymer blends exhibit negative deviations of the glass temperature from values predicted by the free volume or flexible bond additivity rules, suggesting a looser packing within those blends. A reasonable explanation assumes that binary hetero-contact formation within the blend may be accompanied by local interchain orientation contributing consequently to conformational entropy changes. The smaller the induced interchain orientation by hetero-contact formation, the larger the mobility in the neighborhood of the contacts and the probability of related conformational entropy changes, causing an equivalent increase of the “free volume” within the blend, i.e., a corresponding decrease of the blend *T*_g_, which finally can be situated below the values predicted by the additivity rules. Vice versa, the corresponding argument will hold for blends with higher interchain orientation induced by intensive exothermic hetero-contact forces.

## 1. Introduction

Two major models have been proposed for the theoretical interpretation of the glass transition phenomenon characteristic of amorphous polymers. Taking into account the observed kinetic character, the free volume model considers the glass transition essentially as a freeze-in process. Nevertheless, the experimentally observed glass transition shows the characteristics of a thermodynamic second order transition, which are time dependent. The thermodynamic model assumes the existence of the glass as a thermodynamic stable fourth state of matter characterized by a real thermodynamic second order transition, but situated far below the experimentally accessible glass transition. This is supported by the “Kauzmann paradox” [1]. According to the free volume theory [2] the molecular mobility is controlled by the free volume and the glass is considered a frozen metastable state of matter, described by an additional, kinetically controlled internal order parameter [3], and a *P-V-T* equation of state. The thermodynamic theory starts with a *S-V-T* equation of state and the glass is supposed to be a fourth state of matter, characterized by zero conformational entropy [4].

Both models were used to explain the composition dependence of the glass temperature of random copolymers and blends of miscible polymers, assuming additivity of the respective basic properties of the blend components, i.e., either of the specific volumes as Gordon and Taylor have suggested [5], which is equivalent, in fact, to the additivity of the relevant free volumes, as Kovacs has demonstrated [6] or of the flexible bonds, responsible for conformational changes as DiMarzio has assumed [7].

Both additivity models result in the same Gordon-Taylor expression for the composition dependence of the glass temperature of the polymer blend:

$$T_{g}=(w_{1}T_{g_{1}}+Kw_{2}T_{g_{2}})/(w_{1}+Kw_{2})$$ (1)

*T*_g_ is the glass temperature of the blend, whereas *w_i_* are the weight fractions and $T_{g_{i}}$ the glass temperatures of the blend components, the subscript 2 referring to the component with the higher *T*_g_. *K* is a model specific parameter, i.e., *K*_GT_ = (*ρ*_1_/*ρ*_2_)(Δ*α*_2_/Δ*α*_1_) for volume additivity and *K*_DM_ = (*μ*_1_/*r*_1_)/(*μ*_2_/*r*_2_) for flexible bond additivity. In the latter expressions *ρ_i_* are the densities and Δ*α_i_* = (*α*_melt_ − *α*_glass_)*_i_* the increments at *T*_g_ of the expansion coefficients of the blend components. *μ_i_* are the masses and *r_i_* the numbers of flexible bonds of the repeating unit.

A thermodynamic approach to the compositional dependence of the glass temperature of compatible polymer blends has also been suggested by Couchman and Karasz [8]. The approach is based on the supposition of continuity of the thermodynamic excess functions at *T*_g_ and equality of the respective excess functions of mixing of the melt and glass. The problem of this approach is, however, that for volume and enthalpy a linear Gordon-Taylor like expression results, whereas for entropy a logarithmic Gordon-Taylor like expression is obtained. In the respective linear equation for compositional dependence of the blend *T*_g_ based on the enthalpy approach and the logarithmic equation based on the entropy approach the value, of the *K* parameter is related to the ratio of the increments of the heat capacities of the blend components, *K*_CK_ = (Δ*C_p_*_2_/Δ*C_p_*_1_). According to Boyer’s rule, Δ*C_p_T*_g_ = constant [9] the *K* parameter can be substituted in the respective Gordon-Taylor like equations by $K_{\text{CK}}=T_{g_{1}}/T_{g_{2}}$. The problem is, however, that an entropy based logarithmic and an enthalpy based linear Gordon-Taylor like equation are not compatible when applied for the same blend.

Assuming the validity of the Simha-Boyer rule, [10], Δ*αT*_g_ = 0.133 (constant), and neglecting in a first approximation the influence of the mostly very similar densities of the blend components, i.e., considering (*ρ*_1_/*ρ*_2_) ≈ 1, the specific *K*_GT_ parameter for volume additivity can also be substituted by the respective reversed ratio of the glass temperatures of the blend components, $K_{\text{GT}}\approx T_{g_{1}}/T_{g_{2}}$. Introducing this value for *K* in the Gordon-Taylor equation [Eq. (1)] the well known Fox equation results [11].

$$1/T_{g}=w_{1}/T_{g_{1}}+w_{2}/T_{g_{2}}.$$ (2)

Although originally proposed for copolymers, the Fox equation can be considered to be valid in a first rough approximation for supposed additivity of the volume, i.e., of the free-volume of the blend components.

**Fig. 1:**
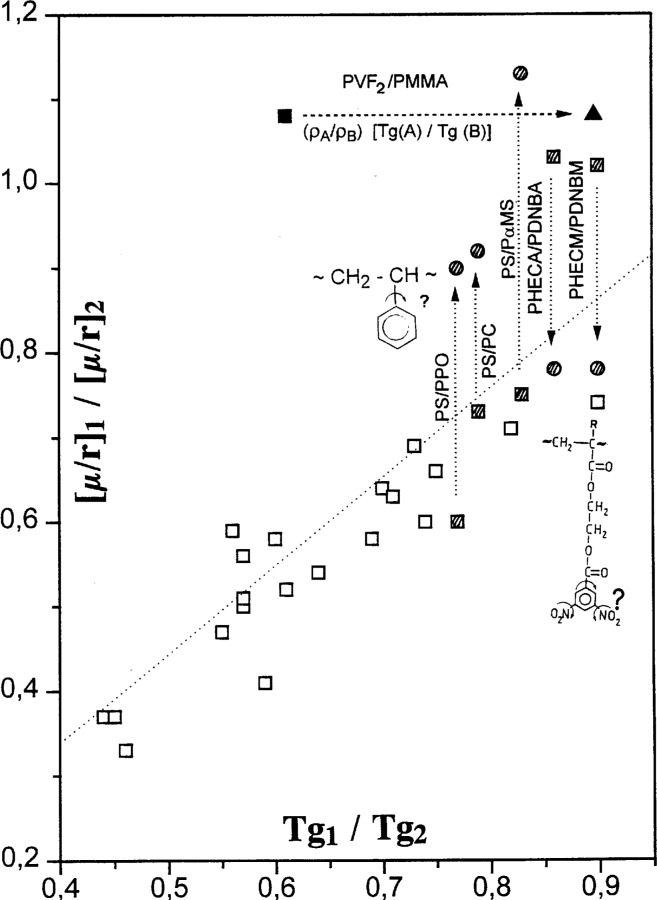
Ratio of the masses/‘flexible’ bonds, [*μ*/*r*]_1_/[*μ*/*r*]_2_ vs ratio of the glass temperatures $T_{g_{1}}/T_{g_{2}}$ of the components of compatible blends.

Surprisingly, both models, i.e., the Fox equation [Eq. (2)] for assumed volume additivity and the DiMarzio equation, i.e., Eq. (1) with *K*_DM_ = (*μ*/*r*)_1_/(*μ*/*r*)_2_ expressed for flexible bond additivity, predict the same composition dependence of the glass temperature of compatible polymer blends as shown by Schneider and DiMarzio [12]. But these additivity-rules-based equations are not able to describe either the experimentally observed positive or negative deviations of the blend *T*_g_ from the predicted additivity values [12,13]. The fact that both models predict the same composition dependence of the blend *T*_g_ is supported by direct correlation between the ratios of the glass temperatures, $T_{g_{1}}/T_{g_{2}}$, and of the masses/flexible bonds of the monomeric units of the blend components, (*μ*/*r*)_1_/(*μ*/*r*)_2_, shown in Fig. 1. Accordingly, it was supposed by Schneider and Di-Marzio [13] that the glass temperature of polymers can be related in a first approximation to the mass/flexible bond of the monomeric unit.

In Fig. 1 are emphasized some of the queries related either with the fact that the influence of possibly very different densities of the polymers is neglected (exemplified for the blend poly(vinilydene floride)/poly-(methyl methacrylate) - PVF_2_/PMMA) or with difficulties encountered in an exact evaluation of the number of flexible bonds of the repeating unit. Thus for instance in poly(styrene), PS, the bond between the planar phenyl ring and backbone can be considered either flexible or not, depending if one assumes or not that two different conformations result depending if the phenyl ring is in plane or out of plane with the backbone. Accordingly two different values for (*μ*/*r*)_PS_ may be used. This is illustrated in Fig. 1 for the blends PS/PPO - (poly(2,3-dimethyl-1,4-phenylene ether), PS/PC - tetramethyl bisphenol-A polycarbonate, and PS/PαMS-poly(α-methyl styrene). Additionally intramolecular or intermolecular interactions may contribute to a stiffening of flexible single bonds or loosening of double bonds. This is exemplified for the probable free electron - π electron interaction in the acceptor polyacrylates or -methacrylates of (β-hydroxyethyl-3,5-dinitrobenzoyl) blended with donor poly-acrylates or -methacrylates of N(2-hydroxyethyl)carbazolyl, i.e. DNBA/PHECA and DNBM/PHECM, respectively. Due to the free electron - π electron interaction the bonds of the dinitrobenzoyl-group, indicated by arrows, can be considered either stiffened or not. A corresponding shift of the values of the (*μ*/*r*)-ratio along the dotted lines would be the consequence.

**Fig. 2:**
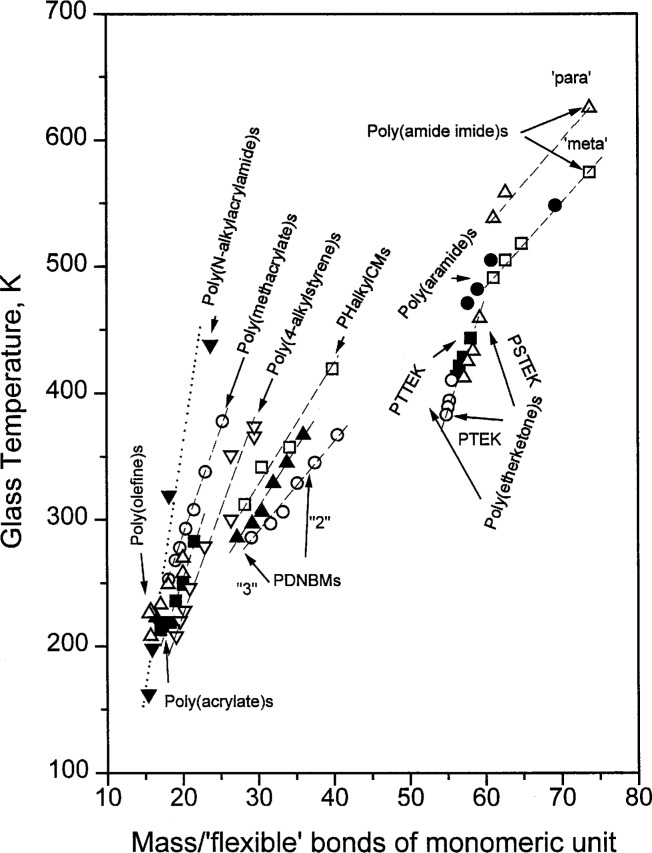
Glass temperature vs mass/‘flexible’ bond of the monomeric unit for different classes of polymers.

These uncertainties concerning an unambiguous determination of the number of flexible bonds of the repeating unit because of possible interactions could be one of the explanations for the observed relatively large scatter of *T*_g_ vs mass/flexible bond of monomeric unit data shown in Fig. 10 of the paper published by Schneider and DiMarzio [13]. Nevertheless, the scatter of the data is not larger than for the correlation between *T*_g_ and conformational flexibility and mass moments of the polymer, recommended by Hopfinger et al. for prediction of polymer glass transition temperature [14]. The assumption that the scatter of the respective *T*_g_ vs mass/flexible bond data is related principally with uncertainties in an exact evaluation of the number of flexible bonds is supported by the observation that the *T*_g_ vs mass/flexible bond rule holds exactly for different classes of polymers as it results from the data illustrated in Fig. 2. Besides literature data for polyolefins, poly(acrylate)s and -(methacrylate)s as well for poly(N-alkylacrylamide)s and for poly(4-alkylstyrene)s [15], are presented our own data for aromatic main chain polymers bearing an increasing number of flexible segments between the aromatic units, i.e., poly(amide imide)s, poly(aramide)s [16] and poly(etherketone)s [17], as well for the polyacceptors, PDNBM, and polydonors, PHalkylCM, containing in the methacrylic side chain an increasing number of methylenes between the methacrylic and the respective electron interacting group [18].

## 2. Concentration Power Equations for the Composition Dependence of the Blend *T*_g_

To account for the effect on *T*_g_ of interactions in polymer blends, empirical concentration second power equations have been proposed in the literature. Jenckel and Heusch [19], for instance, suggested for plasticized polymer blends the expression:

$$T_{g}=w_{1}T_{g_{1}}+w_{2}T_{g_{2}}+b\left(T_{g_{2}}-T_{g_{1}}\right)w_{1}w_{2}$$ (3)

with *b* a parameter which characterizes the solvent quality of the plasticizer. Kwei [20], for his part, extended the Gordon-Taylor equation, introducing in Eq. (1) an additional square concentration term, *qw*_1_*w*_2_, *q* being considered an interaction dependent parameter. Additionally, the *K* parameter is treated as a real fitting parameter of the resulting concentration second power equation:

$$T_{g}=\left(w_{1}T_{g_{1}}+Kw_{2}T_{g_{2}}\right)/\left(w_{1}+Kw_{2}\right)+qw_{1}w_{2}.$$ (4)

Concentration second power equations for the compositional dependence of the blend *T*_g_ were also obtained by DiMarzio [7], by assuming beside flexible bond additivity, the effect of volume changes due to the different specific volumes of the blend components at *T*_g_ and by Kanig [21], who related the changes in interaction energies to the respective Gibbs energies for generating one mole of holes in the equilibrium polymer melt.

Brekner et al. [22] have suggested that the glass transition temperature of compatible polymer blends depends on the free volume distribution and the related conformational mobility, which is controlled by the probability of hetero-molecular contact formation in the mixture due to specific interactions of the components. Applying the lattice theory of regular solutions and supposing that the number of each contact type, both homo- and hetero-contacts, is related with the respective volume fraction of the components, the following concentration second power equation was obtained:

$$\left(T_{g_{2}}-T_{g_{1}}\right)/\left(T_{g_{2}}-T_{g_{1}}\right)=\left(1+K_{1}\right)\phi -K_{1}\phi^{2}$$ (5)

with *ϕ* the volume fraction of the component with the higher *T*_g_. The *K*_1_ parameter is given by the expression $K_{1}=(2E_{12}-\left(E_{11}+E_{22}\right)/\left(T_{g_{2}}-T_{g_{1}}\right)$, *E_ij_* being the respective contact-specific interaction enthalpy. Additivity is thus characterized by *K*_1_ = 0, i.e., the contribution of the intermolecular-hetero contacts is identical with the mean of the binary-homo contacts. The resulting expression for additivity

$$\left(T_{g}-T_{g_{1}}\right)/\left(T_{g_{2}}-T_{g_{1}}\right)=\phi$$ (6)

is identical to the Gordon-Taylor equation [Eq. (1)] rearranged in the form:

$$\left(T_{g}-T_{g_{1}}\right)/\left(T_{g_{2}}-T_{g_{1}}\right)=K_{\text{GT}}w_{2}/\left(w_{1}+K_{\text{GT}}w_{2}\right)=w_{2c}$$ (7)

i.e., the volume fraction, *ϕ*, of the component with the higher *T*_g_ is identical with the weight fraction, *w*_2c_, corrected to account for the differences in density and expansivity of the blend components. Accordingly, Eq. (5) can be expressed as follows:

$$\left(T_{g}-T_{g_{1}}\right)/\left(T_{g_{2}}-T_{g_{1}}\right)=\left(1+K_{1}\right)w_{2c}–K_{1}{w_{2c}}^{2}.$$ (8)

Taking into account the thermodynamic condition of miscibility, Δ*G*_m_ = Δ*H*_m_ − *T*Δ*S*_m_ < 0, and that for polymers the combinatorial entropy of mixing is negligible, i.e., Δ*S*_m_ ~ 0, the enthalpy of mixing has to be exothermic to assure polymer miscibility, i.e., Δ*H*_m_ < 0. That means the energy of hetero-contact interaction has to overcome the energies of homo-contact interactions, i.e., always *E*_12_ > 1/2(*E*_11_ + *E*_22_), for *K*_1_ > 0 in Eq. (8). Thus considering the effect of interactions only, by Eq. (8) exclusive positive deviations from additivity of the blend *T*_g_ can be explained, taking into account that *K*_1_*w*_2c_ > *K*_1_*w*_2c_^2^ for any weight fraction.

To explain negative deviations from additivity the binary contact model was extended to account for the effect of the hetero-contact formation on conformational rearrangements in the immediate molecular neighbor-hood of the binary contacts, accompanied by corresponding conformational entropy changes, see Refs. [22] and [23]. The result is a virial-like concentration third power equation:

$$\left(T_{g}-T_{g_{1}}\right)/\left(T_{g_{2}}-T_{g_{1}}\right)=\left(1+K_{1}\right)w_{2c}-\left(K_{1}+K_{2}\right){w_{2c}}^{2}+K_{2}{w_{2c}}^{3}$$ (9)

where the parameters are expressed by:

$$\begin{array}{l} \;K_{1}=\underset{⇓}{\{[(E_{12-1}+E_{12-2}}-(E_{11-1}+E_{22-2})]\\ \text{energetic interactions, always}>0\\ -\underset{⇓}{\left[(e_{12-2}-e_{12-1})+(e_{11-1}-e_{11-2})]\right\}}/(T_{g_{2}}-T_{g_{1}})\\ \text{effects induced by conformational changes} \end{array}$$ (10)

$$\begin{array}{l} \;K_{2}=\{[(2e_{12-1}-(e_{11-12}+e_{22-1})]\\ \;-\underset{⇓}{\left[2e_{12-2}-(e_{22-2}+e_{22-1})\right]}/\underset{⇓}{\left(T_{g_{2}}-T_{g_{2}}\right)}.\\ \text{energetic effects induced by conformational changes} \end{array}$$ (11)

*K*_1_ depends essentially on the difference between the interaction energies of hetero- and homo-contacts, but it includes also the effects of the induced conformational changes in the neighborhood by hetero-contact formation. *E*_11−1_ = *E*_11_ + *e*_11_ and *E*_22−2_ = *E*_22_ + *e*_22_, respectively, characterize the behavior of the pure components, whereas the hetero-contact formation is considered either in an exclusive environment of component 1 or 2, i.e., *E*_12−1_ = *E*_12_ + *e*_12−1_ or *E*_12−2_ = *E*_12_ + *e*_12−2_. The first rectangular bracket of *K*_1_ accounts consequently for the energetic effects resulting from the substitution of one homo-contact by a hetero-contact in the pure homo-environment. The second rectangular bracket of the parameter *K*_1_ includes by the first difference (*e*_12−1_ − *e*_12−2_) the conformational determined effects of substitution in the surroundings of the binary hetero-contact of one neighbor 1 by a neighbor 2. The second difference (*e*_11−2_ − *e*_11−1_) is the consequence of the asymmetry of the concentration power Eq. (9) because of choosing as the effective variable the volume fraction, i.e., the corrected weight fraction, *w*_2c_, of the stiffer component with the higher *T*_g_. This asymmetry is reflected in the difference between the parameters *K*_1_ and *K*_2_.

$$\begin{array}{l} K_{1}-K_{2}=\{[(E_{12-1}+E_{12-2}-(E_{11-1}+E_{22-2})]\\ -\left[\left(e_{12-1}-e_{12-2}\right)+\left(e_{22-2}-e_{22-1}\right)\right]\}/\left(T_{g_{2}}-T_{g_{1}}\right). \end{array}$$ (12)

Comparing Eq. (12) with Eq. (10) for *K*_1_ shows that they differ exclusively by the contributions of conformational changes, comprised in the respective second rectangular brackets. In the first difference term included in the brackets in fact only the order of the conformational influences of the homo-environments on hetero-contacts is inversed. The second differences corroborate effectively the asymmetry of the respective expressions. Thus in Eq. (10) the influence of substitution of one component 1 by component 2 in the pure environment of component 1 is considered, whereas in Eq. (12) the opposite substitution is taken into account.

In Eq. (11) for the parameter *K*_2_ the first difference represents the effects induced by the conformational changes due to hetero-contact formation in a predominately component 1, whereas the second difference refers to the same effect of hetero-contact formation in the predominantly component 2 environment. The parameter *K*_2_ can thus be assumed to be characteristic for energetic influences on the binary contact interaction due to entropy changes induced by the conformational rearrangements caused by hetero-contact formation in the binary compatible polymer blend.

Unfortunately, the parameters *K*_1_ and *K*_2_ are not yet accessible by other means but only via fitting of the concentration third power Eq. (9) to experimental *T*_g_ vs concentration data of compatible polymer blends. Thus, it is not possible to separate the compositional dependent enthalpic from the conformational induced entropic contributions to the glass temperature of polymer blends. Nevertheless, by using the concentration power Eq. (9), it is possible to explain additionally the observed negative deviations from additivity of the blend *T*_g_, because depending on the conformational entropy contributions, both the parameters *K*_1_ and *K*_2_ can adapt not only positive, but also negative values.

## 3. Results and Discussions

In Table 1 are presented the values of the fitting parameters of the virial-like concentration power Eq. (9) for the compositional dependence of the glass temperature of some representative compatible blends of homo-polymers. For the evaluation of the fitting parameters both literature and our own *T*_g_ vs composition data of binary polymer blends were used. The blends are arranged in Table 1 according to decreasing values of the *K*_1_ parameter. Taking into account the values of *K*_1_ and of the difference of the (*K*_1_ − *K*_2_) parameters, the blends can be arranged into five major classes which show different specific *T*_g_ vs composition curves.

**Fig. 3:**
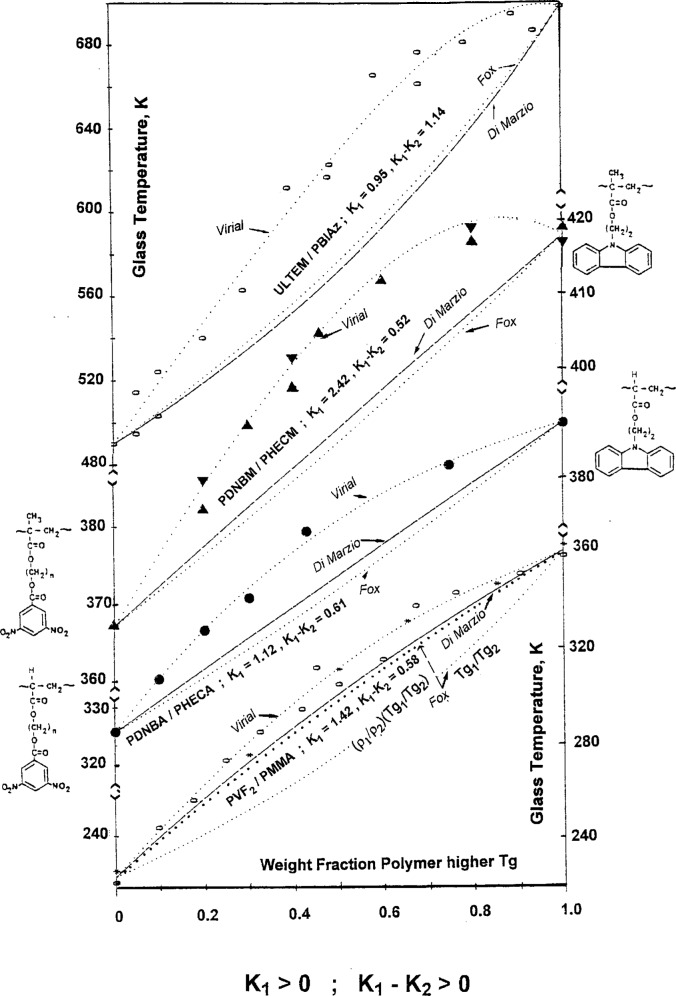
*T*_g_ vs weight fraction of the polymer blend component with the higher $T_{g_{1}}$ for polymer blends with positive deviations of the *T*_g_ from additivity.

In the first class are included the blends characterized by positive values of both *K*_1_ and the (*K*_1_ − *K*_2_) difference. Depending on the values of the parameters the blends show all more or less pronounced positive deviations from additivity of the blend *T*_g_ as it results from the data presented in Fig. 3. Beside the curves fitted according to the virial like concentration power Eq. (9) are shown both the curves predicted by the volume (Fox—dotted lines) and by the mass/flexible bond additivity model (DiMarzio—dashed lines). Except for PVF_2_/PMMA, in Fig. 3 the same *T*_g_ behavior is predicted by both additivity models for the three other blends shown. In fact, of all studied polymer blends, the PVF_2_/PMMA blend is the only one which shows different behavior for volume and mass/flexible bond additivity. But taking into account the very different densities of the two homopolymers, a corrected Fox—volume additivity model (see thick dotted line) predicts almost the same behaviour as the mass/flexible bond model.

Values of the *K*_2_ parameter very different from zero are characteristic for asymmetric *T*_g_ vs blend composition curves. For absolute values of |*K*_2_| > |*K*_1_|, the difference of the fitting parameters, (*K*_1_ − *K*_2_), has always the opposite sign from *K*_1_ and the corresponding *T*_g_ vs blend composition curves are S-shaped.

**Fig. 4:**
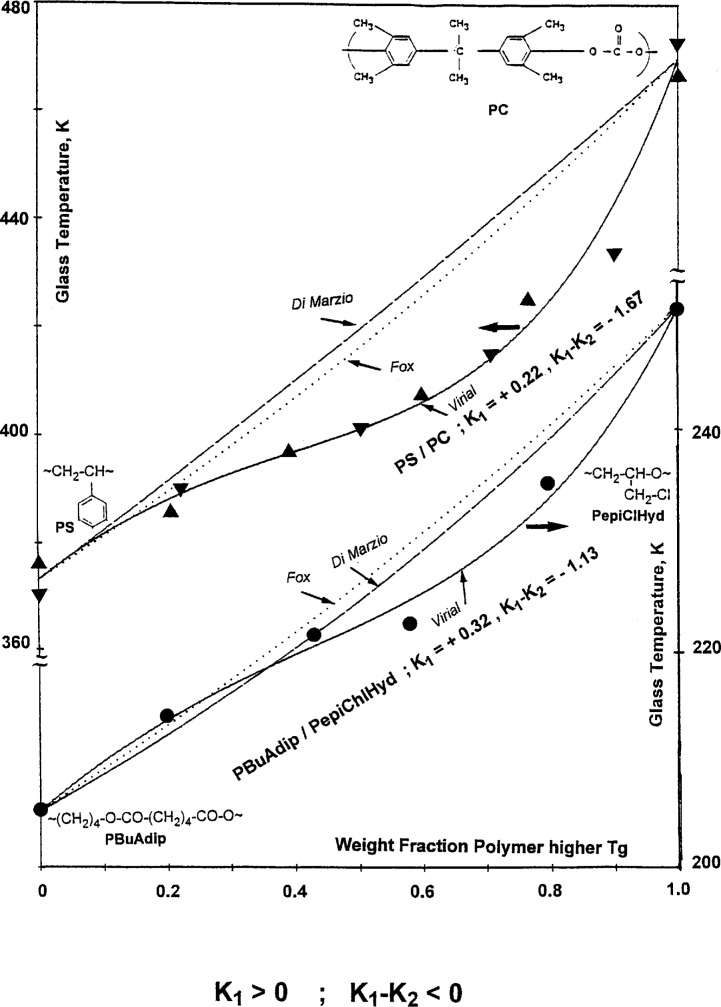
S-shaped *T*_g_ vs composition curves for polymer blends characterized by the parameters of the concentration power equation *K*_1_ > 0; (*K*_1_ − *K*_2_)<0.

For the pair *K*_1_ > 0 and (*K*_1_ − *K*_2_) < 0, the respective *T*_g_ vs composition curves show generally positive deviations from additivity for higher concentrations of component 1 with the lower $T_{g_{1}}$ and negative deviations in the high concentration range of the component 2 with the higher $T_{g_{2}}$. This is illustrated in Fig. 4 for the blends PS/PC and poly(butylene adipate)/poly(epichlorohydrine) - PBuAdip/PepiClHyd -, respectively. The larger the difference between the positive *K*_1_ and the negative (*K*_1_ − *K*_2_) values, the larger the negative deviations from additivity of the blend *T*_g_ in the high concentration range of the component 2 with the higher $T_{g_{2}}$.

**Fig. 5:**
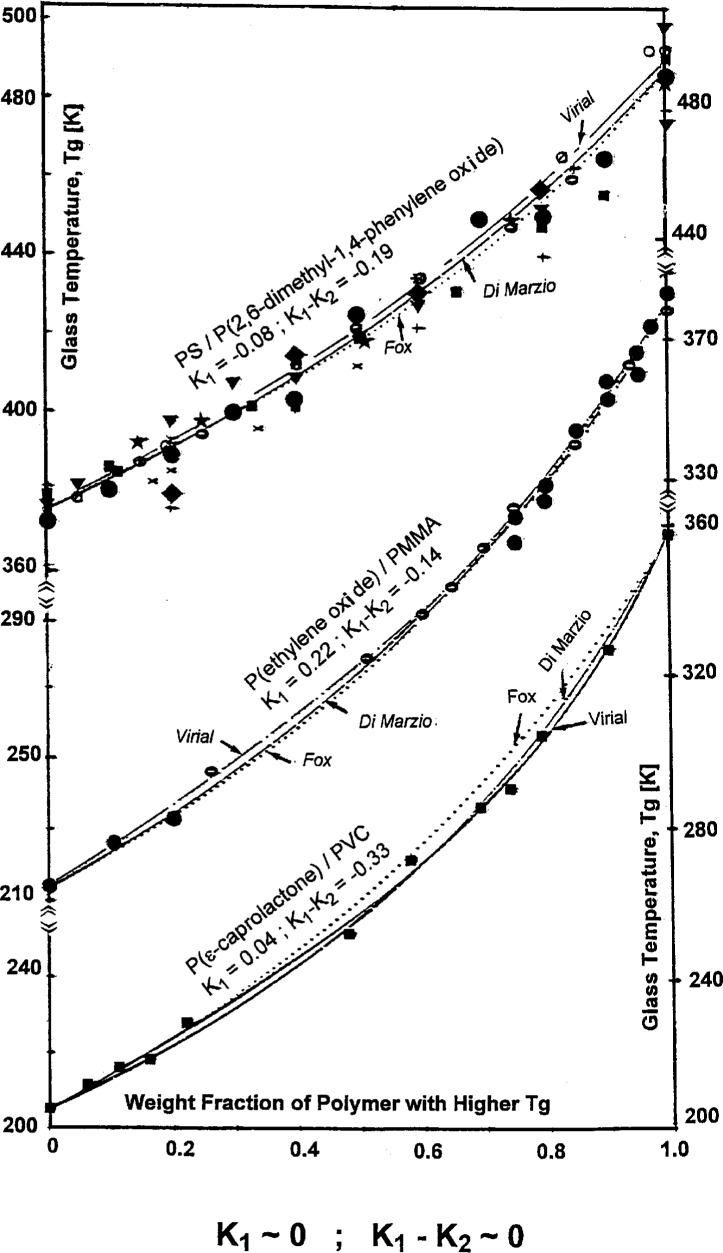
*T*_g_ vs composition curves for polymer blends with almost additive *T*_g_.

Additivity of the blend *T*_g_ is observed for values of the two fitting parameters, *K*_1_ and *K*_2_, of the concentration power equation ranged between +0.4 and −0.4. In this case the compositional dependence of the glass temperature is predicted by both the volume additivity (Fox) and flexible bond additivity (DiMarzio) models. This is demonstrated in Fig. 5 for the blends: PS/PPO, poly(ethylene oxide)/poly(methyl methacrylate) - PEO/PMMA - and poly(ϵ-caprolactone)/poly(vinyl-chloride) − PϵCL/PVC.

Taking into account that for volume additivity the *K* parameter of the Gordon-Taylor equation can be substituted in a first approximation by $T_{g_{1}}/T_{g_{2}}$, the *T*_g_ vs composition curves are always slightly concave.

**Fig. 6:**
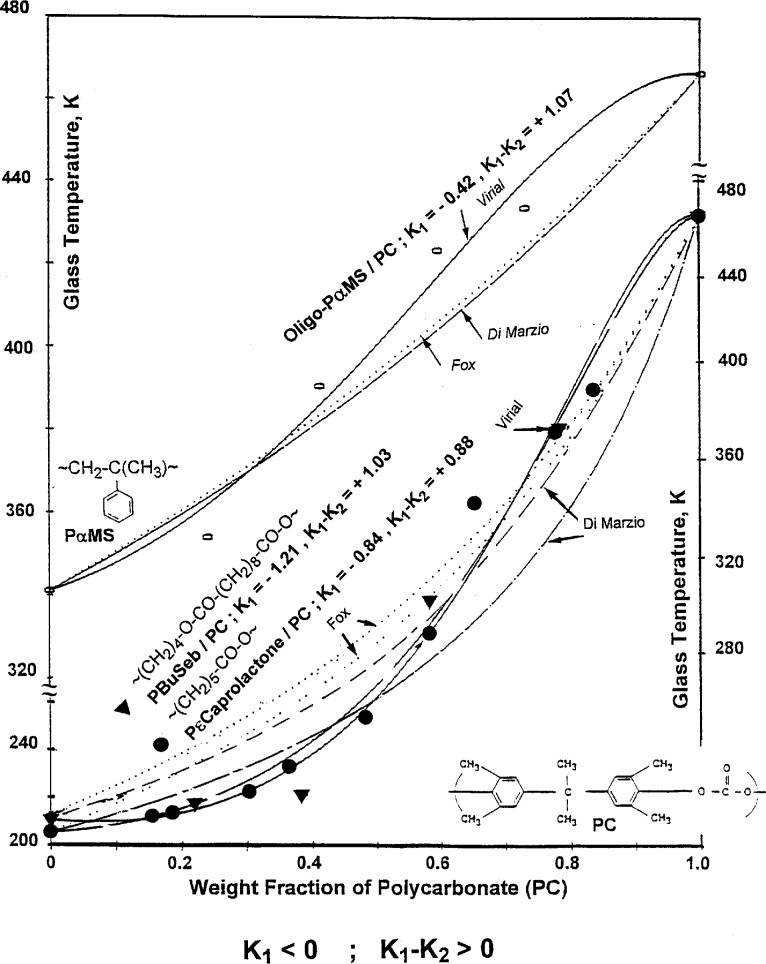
S-shaped *T*_g_ vs composition curves for polymer blends characterized by the parameters of the concentration power equation *K*_1_ < 0 and (*K*_1_ − *K*_2_) > 0.

The form of the S-shaped blend *T*_g_ vs composition curves is reversed if *K*_1_ < 0 and (*K*_1_ − *K*_2_) > 0 i.e., they show negative deviations in the high concentration range of the component 1 with the lower $T_{g_{1}}$ and positive deviations in the high concentration range of the component 2 with the higher $T_{g_{2}}$. This is illustrated in Fig. 6 for the blends of PC with PαMS, poly(butylene sebacate) -PBuSeb - and poly(ϵ-caprolactone).

**Fig. 7:**
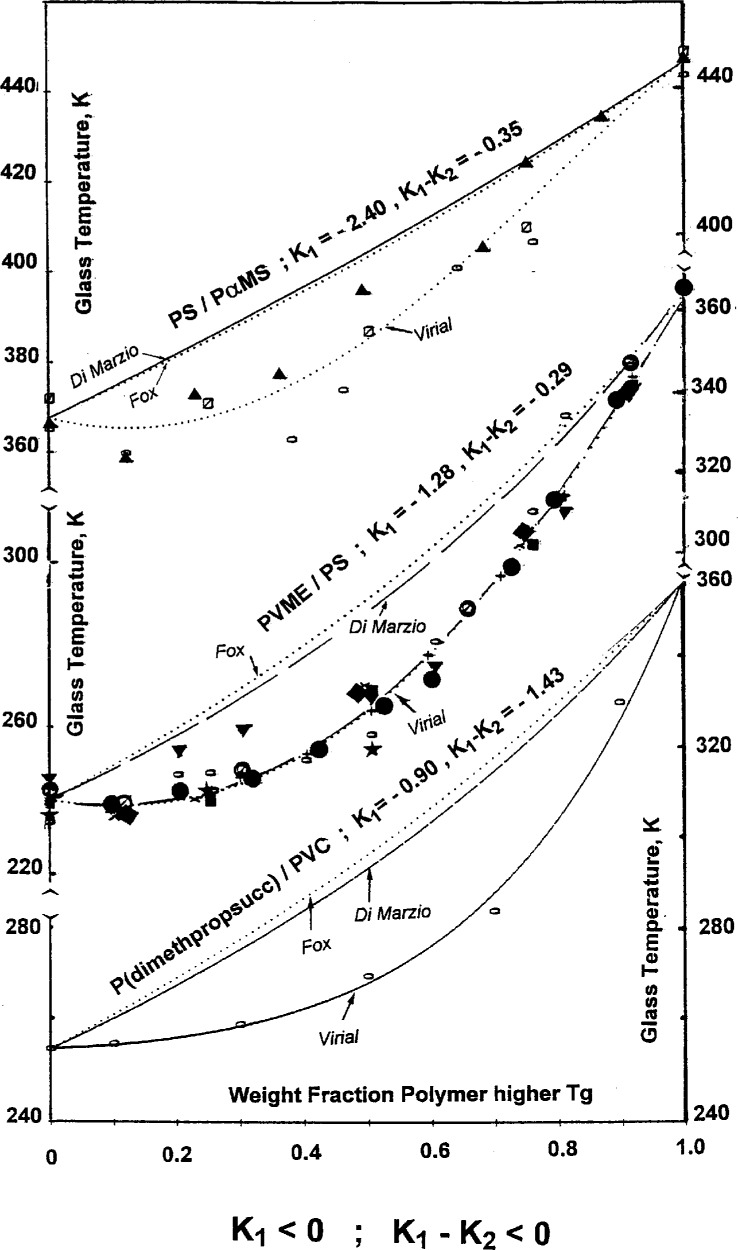
*T*_g_ vs composition curves for polymer blends with negative deviations of the *T*_g_.

Finally, negative deviations of additivity are characterized by negative values of both the *K*_1_ parameter and the difference, (*K*_1_ − *K*_2_), between the fitting parameters of the concentration power equation—see Fig. 7. Again the two additivity models predict the same behavior and are not able to describe experimental *T*_g_ vs composition data.

**Fig. 8:**
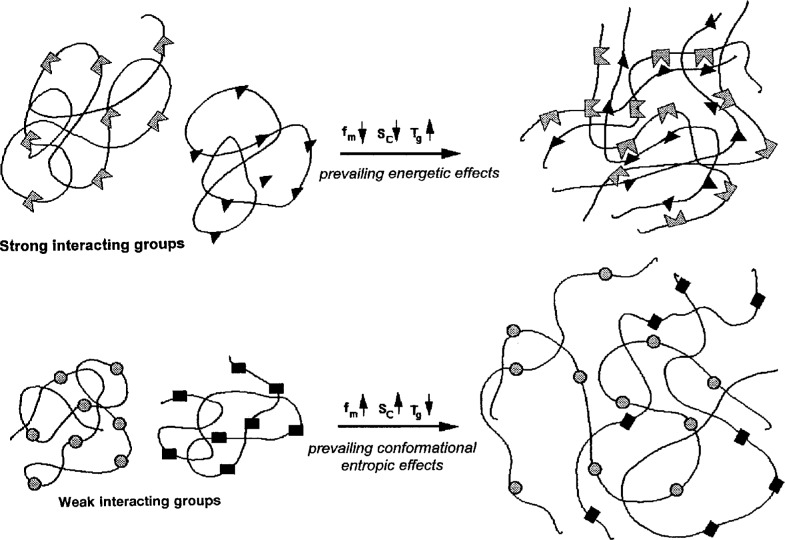
Models for polymer blends with prevailing energetic local ordering effects or predominant conformational entropy contributions to the interaction energy of binary hetero-contacts.

Considering the nature of the interaction energies needed to assure compatibility of polymers it may be assumed that the stronger the interaction energy, the larger the probability of an induced neighboring hetero-contact formation accompanied by a local interchain ordering, restricting the conformational mobility in the neighborhood of the hetero-contacts. As a consequence, both the conformational entropy and the free volume are diminished in the blend. This is reflected by an increase of the blend glass temperature above the temperature predicted by additivity rules. Accordingly, polymer blends characterized by strong interactions will show positive deviations from additivity of the blend glass temperature, like the PDNBM/PHECM blend bearing strong electron donor—electron acceptor interaction. Blends with weaker interaction energies, as for instance the π-πelectron interaction in the PS/PαMS blend, will show less or no local ordering due to hetero-contact formation and thus an enhanced conformational mobility. Accordingly both conformational entropy and free volume will increase, whereas the blend *T*_g_ shows negative deviations from additivity. These possible opposite effects of local ordering by hetero-contact formation are sketched in Fig. 8.

**Fig. 9:**
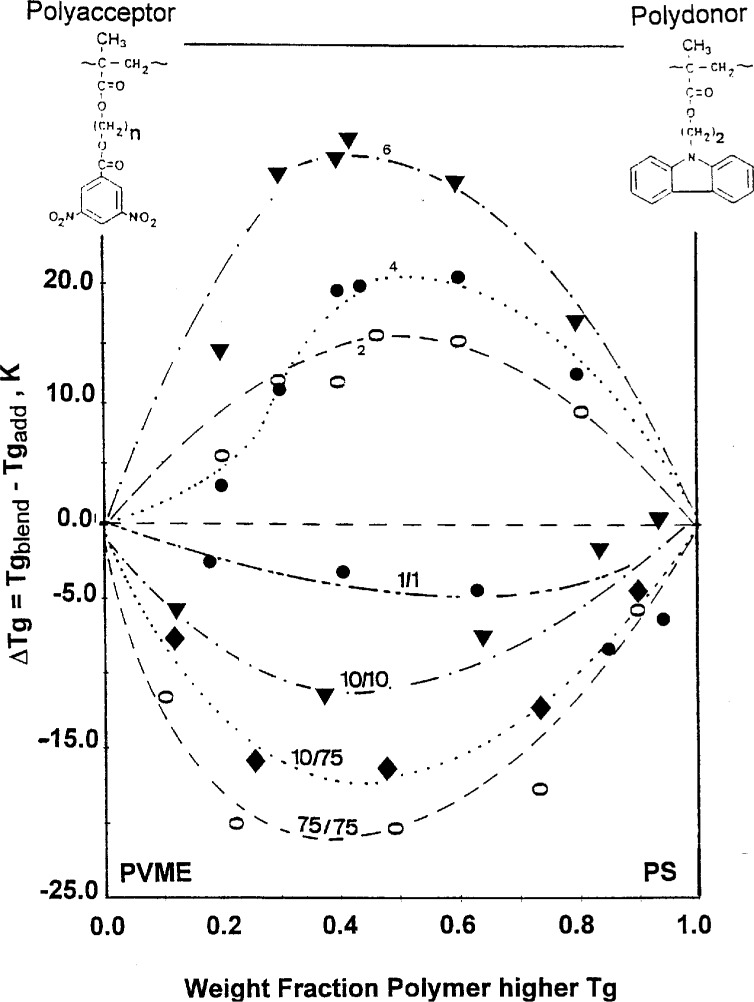
Dependence of the deviation of the blend *T*_g_ from additivity—for the strong interacting polyacceptor/poly-donor blends of PDNBM/PHECM on the spacer length between the acceptor group and methacrylic backbone of PDNBM; the figures on the curves indicate the number of methylenic units in the spacer of PDNBM—for the weaker interacting PVME/PS blends on the molecular weight of the blend components; the figures on the curves indicate the molecular weights in thousands of the components of the PVME/PS blends.

The assumed local ordering effect of hetero-contact interaction is supported by the data presented in Fig. 9. For the strong electron donor—electron acceptor interaction the probability of neighboring hetero-contact formation by charge transfer complexation of the acceptor poly(β-hydroxyalkyl-3,5-dinitrobenzoyl methacrylate) with the donor poly-[N(2-hydroxyethyl)carbazoyl methacrylate] is additionally enhanced by the increasing spacer length between the interacting dinitrobenzoyl electron acceptor group and the methacrylic backbone as it results from Fig. 9 using data published in [18]. For weaker interactions, on the contrary, the probability of hetero-contact formation is decisively influenced by the molecular weight of the blend components. The higher the molecular weight, the more probable the coiling of the polymers and thus the less accessible are the interacting groups for a random hetero-contact formation. Accordingly, the negative deviation of the blend *T*_g_ from additivity will decrease with increasing molecular weight of the blend components. This is illustrated in Fig. 9 for PVME/PS blends, using data published by Schneider and Leikauf [38].

It may thus be supposed that the weaker the interaction energy between the blend components, the less probable the local ordering effect of hetero-contact formation on the immediate environment allowing additional conformational rearrangements. As a consequence, the conformational entropy contribution to the polymer miscibility increases. These prevailing conformational entropy effects will contribute to an increase of the free volume and a corresponding decrease of the blend *T*_g_. Predominant energetic interaction effects, on the contrary, will cause an increase of the glass transition due to the denser packing in the blend because of decreasing mobility and free volume conditioned by the local ordering effect of hetero-contact formation. It may thus be assumed that additivity in polymer blends is the consequence of compensation of energetic interaction effects and conformational entropy contributions to polymer miscibility. The situation concerning the balance between conformational entropy and enthalpy contributions and its influence on the glass temperature of miscible polymer blends is analogous to the *θ*-point situation in polymer solutions, where the balance of enthalpic and entropic contributions depends on the strength of the specific polymer-solvent interaction.

Taking into account that enthalpy of mixing of polymers is connected in a first approximation to the mutual solubility of the components, subsequently it is attempted to correlate the fitting parameters of the concentration power equation with the difference between the solubility parameters of the blend components, (*δ*_2_ − *δ*_1_). The solubility parameters were estimated according to the group contribution method by using the sets of group constants recommended by Small based on van Krevelen’s analysis [40].

**Fig. 10:**
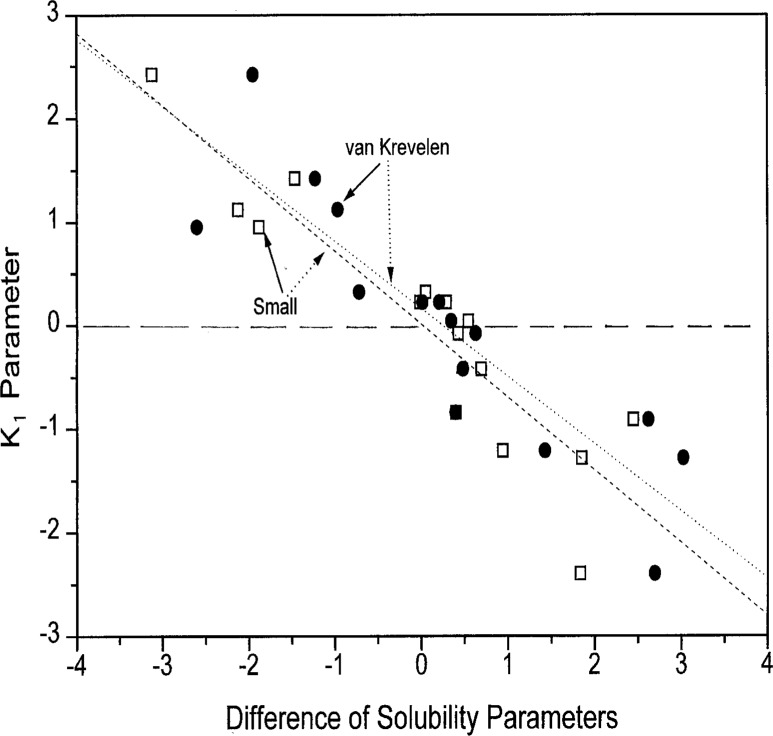
Correlation between the *K*_1_ parameter of the concentration power equation and the difference (*δ*_2_ − *δ*_1_) of the solubility parameters of the blend components.

Although the *K*_1_ parameter, Eq. (10), comprises not only energetic contributions to miscibility, but also contains conformational entropic effects to the binary hetero-contact interaction energy, the correlation with the difference between the solubility parameters of the blend components is surprisingly good as is seen in the data presented in Fig. 10. Predominant energetic interaction effects, characterized by positive values of *K*_1_ are connected with negative values of the difference of the solubility parameters. For preponderant conformational entropic contributions, indicated by negative values of *K*_1_, on the contrary, positive values of the difference between the solubility parameters are specific. For additivity, both *K*_1_ and the difference between the solubility parameters show values near zero.

**Fig. 11:**
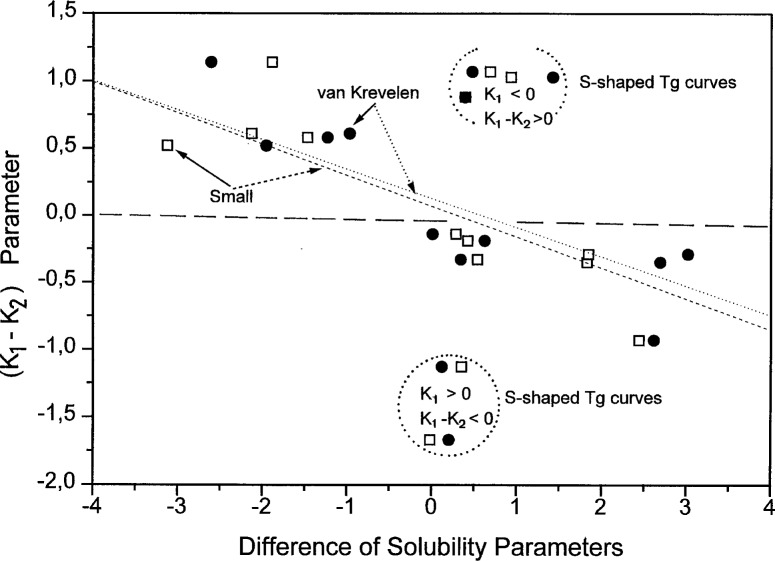
Correlation between the difference (*K*_1_ − *K*_2_) of the concentration power equation and the difference (*δ*_2_ − *δ*_1_) of the solubility parameters of the blend components.

**Fig. 12:**
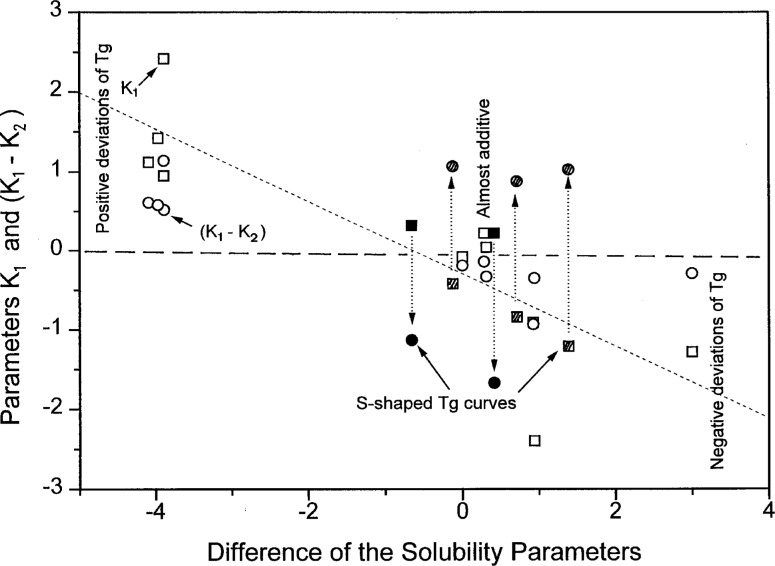
Parameters of the concentration power equation vs difference of the solubility parameters of compatible polymer blends.

Similar dependences are observed for the correlation between the differences of the (*K*_1_ − *K*_2_) parameters and of the solubility parameters, as shown in Fig. 11. The scatter of the data is, however, larger for the (*K*_1_ − *K*_2_) parameter difference. It is supposed that this different behavior is related to the asymmetry of the two expressions, Eqs. (10) and (12), respectively, which comprise beside the energetic contribution to miscibility, different shares of the conformational entropy effects on the binary hetero-contact interaction energies. The difference is evident mainly for the S-shaped *T*_g_ vs composition curves, and is related with the fact that for S-shaped curves the absolute values of the parameter *K*_2_ are larger then those of the *K*_1_ parameter, causing an inversion of the sign for the (*K*_1_ − *K*_2_) difference, as is evidenced by the data shown in Fig. 12.

## Figures and Tables

**Table 1:** Parameters of the concentration power equation applied to the glass temperature of compatible polymer blends $\left(T_{g}-T_{g_{1}})/(T_{g_{2}}-T_{g_{1}})=(1+K_{1})w_{2c}-(K_{1}+K_{2}){w_{2c}}^{2}+K_{2}{w_{2c}}^{3}w_{2c}=Kw_{2}/(w_{1}+Kw_{2}\right)$; $K=T_{g_{1}}/T_{g_{2}}$

Blend	$K=T_{g_{1}}/T_{g_{2}}$	*K*_1_	*K*_2_	*K*_1_ − *K*_2_	Ref.
Blend *T*_g_—positive deviations from additivity; Δ*T*_g_ > 0 characteristic of prevailing enthalpic effects due to strong interactions *K*_1_ and *K*_1_ − *K*_2_ >0; see Fig. 3					

PDNBM/PHECM	0.90	2.42	1.90	0.52	[24]
PDNBA/PHECA	0.86	1.12	0.51	0.61	[25]
P(vinylidene flouride)/PMMA	0.61	1.42	0.84	0.58	[26]
ULTEM^R^/P(Benzimideazole)	0.70	0.95	−0.19	1.14	[27]

S-shaped *T*_g_ vs composition curves *K*_1_ > 0 and *K*_1_ − *K*_2_ < 0; see Fig. 4					

P(butylene adipate)/P(epichlorohydrine)	0.82	0.32	1.45	−1.13	[28]
PS/PC	0.79	0.22	1.89	−1.67	[29],[30]

Blend *T*_g_—almost additive; Δ*T*_g_ ~ 0 |*K*_1_| and |*K*_2_| < 0.5; see Fig. 5					

P(ethylene oxide)/PMMA	0.56	0.22	0.36	−0.14	[31]
P(ϵ-caprolactone)/PVC	0.57	0.04	0.37	−0.33	[32]
PS/P(2,6-dimethylphenylene oxide)	0.77	−0.08	0.11	−0.19	[33]

S-shaped *T*_g_ vs composition curves *K*_1_ < 0 and *K*_1_ − *K*_2_ > 0; see Fig. 6					

P(α-methylstyrene)/PC	0.73	−0.42	−1.49	1.07	[29]
P(ϵ-caprolactone)/PC	0.44	−0.84	−1.72	0.88	[34],[35]
P(butylene sebacate)/PC	0.45	−1.21	−2.24	1.03	[34]

Blend *T*_g_—negative deviations from additivity; Δ*T*_g_ < 0 characteristic of prevailing confromational entropic effects for weaker interactions *K*_1_ and *K*_1_ − *K*_2_ < 0; see Fig. 7					

Pdimethpropsucc/PHEBA	0.69	−0.91	0.02	−0.93	[36]
P(vinylmethyl ether)/PS	0.65	−1.28	−0.99	−0.29	[37],[38]
PS/PαMS	0.83	−2.40	−2.05	−0.35	[39]

PDNBM resp. PDNBA - poly(ω-hydroxyethyl-3,5-dinitrobenzoyl methacrylate) resp. -acrylate)

PHECM resp. PHECA - poly[N-(2-hydroxyethyl)carbazolyl methacrylate) resp. -acrylate)

ULTEM^R^ - poly{[2,2’-bis(3,4-dicarboxyphenoxy)phenylpropane]-2-phenylene-bisimide}

PC - tetramethyl-bisphenol-A-polycarbonate; PHEBA - polyhydroxyether of bisphenol-A

P(dimethpropsucc) - poly(2,2′ dimethyl-1,3-propylene succinate).
